# Ti-O-O coordination bond caused visible light photocatalytic property of layered titanium oxide

**DOI:** 10.1038/srep29049

**Published:** 2016-06-28

**Authors:** Xingang Kong, Chaobin Zeng, Xing Wang, Jianfeng Huang, Cuiyan Li, Jie Fei, Jiayin Li, Qi Feng

**Affiliations:** 1 School of Materials Science and Engineering, Shaanxi University of Science and Technology, Weiyang, Xi’an, Shaanxi 710021, P. R. China; 2 Department of Advanced Materials Science, Faculty of Engineering, Kagawa University, 2217-20 Hayashi-cho, Takamatsu-shi, 761-0396 Japan

## Abstract

The layered titanium oxide is a useful and unique precursor for the facile and rapid preparation of the peroxide layered titanium oxide H_1.07_Ti_1.73_O_4_·nH_2_O (HTO) crystal with enhanced visible light photoactivity. The H_2_O_2_ molecules as peroxide chemicals rapidly enter into the interlayers of HTO crystal, and coordinate with Ti within TiO_6_ octahedron to form a mass of Ti-O-O coordination bond in the interlayers. The introduction of these Ti-O-O coordination bonds result in lowering the band gap of HTO, and promoting the separation efficiency of the photo induced electron–hole pairs. Meanwhile, the photocatalytic investigation indicates that such peroxide HTO crystal has the enhanced photocatalytic performance for RhB degradation and water splitting to generate oxygen under visible light irradiating.

The pure titanium oxide (TiO_2_) as photocatalytic materials, mainly in the UV light region, have the photocatalytic activity due to its wide band gap (3.2 eV), which limits their optical absorption in the UV region (less than 5% of the entire solar energy)^1^,^2^. To increase the optical absorption extent of TiO_2_ under sunlight, there have been efforts to modify the energy band structure of TiO_2_ by doping metal or nonmetal impurities that generate donor or acceptor states in the band gap^3^,^4^,^5^. The metal doping could lead to the formation of secondary impurities which could deteriorate the photocatalytic performance. In the case of nonmetal doping, excess anionic doping (such as N, C, S) could promote the formation of oxygen vacancies, which reduces the photoactivity by enhancing electron–hole recombination^6^,^7^. However, very recently oxygen richness in TiO_2_ (O_2_^2−^ self-doping) have found the impetus for inducing optical properties and thereby reinforcing effective photocatalysis^8^,^9^,^10^.

At present, the oxygen-rich TiO_2_ is mainly prepared by the thermal decomposition of peroxo-titania complex xerogel, in which the H_2_O_2_ solution is usually used as the oxygen rich chemicals^8^,^9^. We all know that the H_2_O_2_ solution easily react with titanium tetrachloride (liquid)^11^, titanium sulfate^12^, titanium alkoxides (liquid)^9^,^13^ or amorphous H_2_TiO_3_ powder (hydrous TiO_2_·nH_2_O)^14^,^15^,^16^, to generate the soluble peroxo titanate complex under the room temperature. However, the crystalline TiO_2_ is simply treated by H_2_O_2_ to not produce the peroxide TiO_2_ crystal or the soluble peroxide titanate complex, and to form the Ti-O-O coordination bonds in the face of TiO_2_ crystal^17^,^18^. This is due to the solid structure of TiO_2_ crystal, resulting in that the H_2_O_2_ molecules cannot enter into the inward of TiO_2_ crystal.

The layered titanium oxide, that is an open structure crystal which is composed of TiO_6_ layers and interstitial hydrated H^+^ ions, is also the wide band gap semiconductor^19^,^20^,^21^,^22^,^23^,^24^. Some researches focus on the photocatalytic property of the layered titanium oxide modified, for example Pt@H_2_Ti_4_O_9_^19^, PAN/H_x_Ti_2-x/4_□_x/4_O_4_^20^, H_0.8_Fe_0.4_Ti_1.6_O_4_^21^, CrO_x_-Ti_1.83_O_4_^22^, TBA_2_Ti_4_O_9_^23^, In this study we use the open structure of the layered titanium oxide H_1.07_Ti_1.73_O_4_·nH_2_O (HTO) crystal, and tactfully introduce the H_2_O_2_ molecules to the inward of HTO crystal, to form the peroxide layered titanium oxide crystal by *in situ* coordination reaction. The peroxide HTO crystal can effectively absorb the visible light of the solar spectrum, and presents the enhanced photocatalytic performance for the RhB degradation under visible light irradiating.

## Result and Discussion

**Figure 1:**
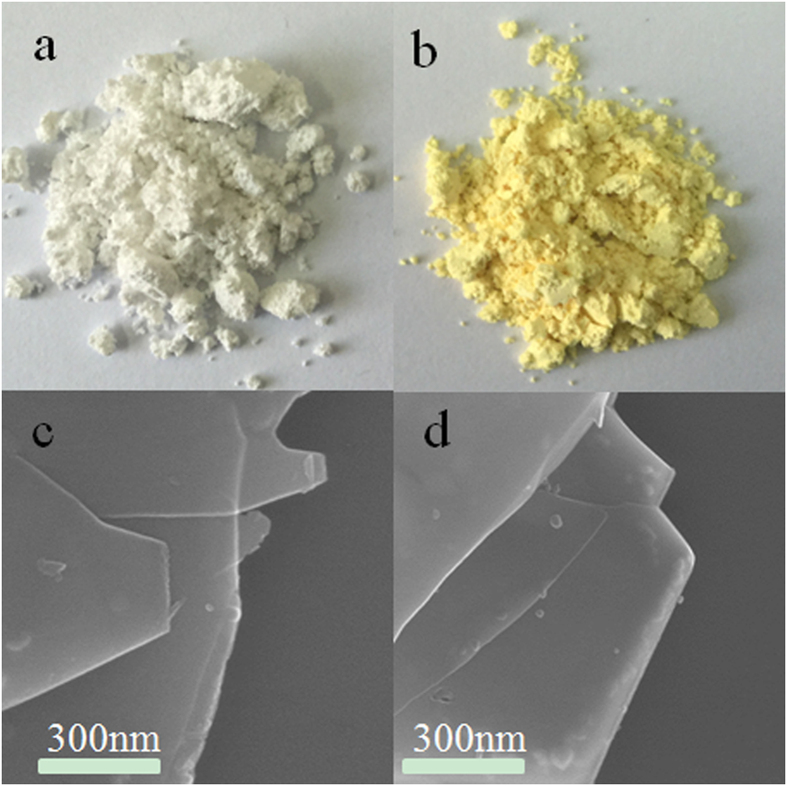
Photo(**a,b**) and FE-SEM(**c,d**) of the HTO crystals (**a,c**)before and (**b,d**)after the treatment in H_2_O_2_ solution, respectively.

It is well known that Ti(IV) easily occurs coordination reaction with hydrogen peroxide H_2_O_2_ to form Ti-hydroperoxide species with yellow^25^. In fact, TiO_2_ crystals is simply placed into the H_2_O_2_ solution, we cannot observe the color change of TiO_2_ crystals with the unaided eye. However, it is interesting that the color of layered titanium oxide HTO crystal quickly changes into yellow from white when it meet with H_2_O_2_ solution (Fig. 1a,b), and the H_2_O_2_ solution remains colorless and transparent. The FE-SEM images display that the H_2_O_2_ treated HTO crystal still possesses the platelike shape with smooth surface (Fig. 1c,d). These indicate that the HTO crystal cannot corrode or dissolve in the H_2_O_2_ solution, and its platelike morphology and microstructural are not influenced by H_2_O_2_. The phenomenon of the HTO color change should be due to the formation of the Ti-O-O coordination bonds.

**Figure 2:**
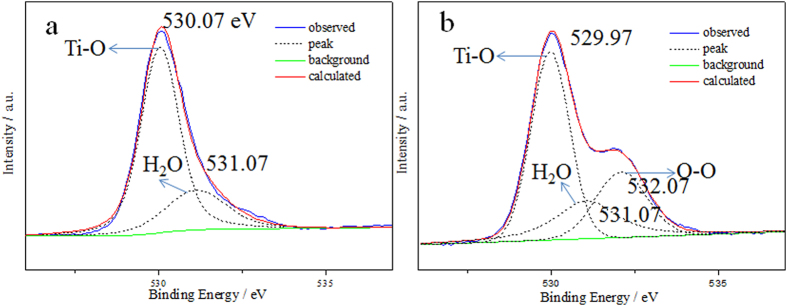
XPS spectra of O1s for (**a**) the HTO crystal and (**b**) the H_2_O_2_ treated HTO crystal.

To confirm whether the existence of the Ti-O-O coordination bonds in the H_2_O_2_ treated HTO crystal, XPS is firstly performed to investigate the H_2_O_2_ treated HTO crystal. In the overall XPS spectrum of the HTO crystal and the H_2_O_2_ treated HTO crystal (Supplementary Fig. S1), the binding energies were calibrated for specimen charging by referencing the C 1s to 284.6 eV. It is found that no peaks of other elements except C, O and Ti were observed in the survey spectrum. The Ti 2p XPS spectra are almost identical for both the pure HTO and the H_2_O_2_ treated HTO (Supplementary Fig. S2), which indicates that Titanium of the HTO crystals before and after the treatment by H_2_O_2_ have a similar bonding environment and are still tetravalence^26^. The O1s spectrum of the HTO crystal is fitted to two peaks at 530.07 and 531.07 eV, which could be respectively assigned to O^2−^ of Ti-O bond within HTO and H_2_O^9^,^27^. In compare with O1s spectrum of HTO, the one of the H_2_O_2_ treated HTO crystal has significant change which a distinct shoulder is observed at 531.9 eV. Peak separation of the O1s spectrum clearly shows three kinds of oxygen with binding energies of 529.97, 531.07 and 532.07 eV (Fig. 2b). The peak located at 529.97, 531.07 and 532.07 eV could be respectively ascribed to O^2−^ of Ti-O bond and H_2_O, and O^1−^ of H_2_O_2_^14^,^28^, which suggest the presence of O_2_^2−^ in the H_2_O_2_ treated HTO crystal.

**Figure 3:**
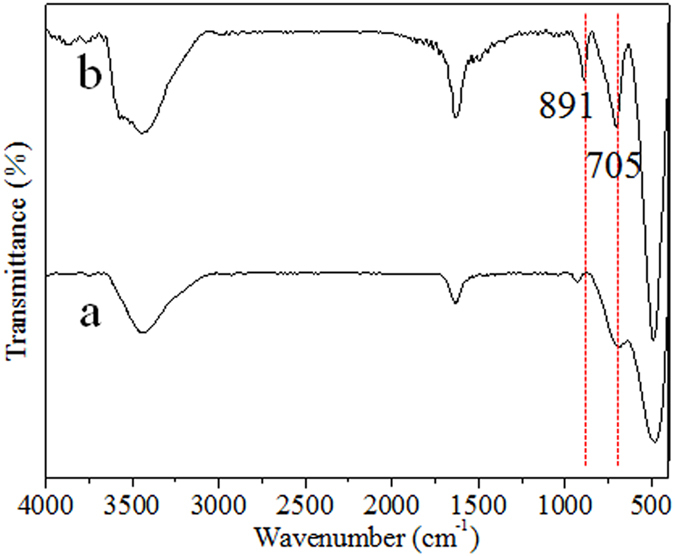
FTIR spectrums of the HTO crystals (**a**) before and (**b**) after the treatment in H_2_O_2_ solution, respectively.

Figure 3 shows the FTIR spectrums of the pure HTO and the H_2_O_2_ treated HTO. The broad bands at 3447 cm^−1^ and 1630 cm^−l^ are assigned to the fundamental stretching and bending vibrations of H_2_O or H_3_O^+^, respectively^29^. The strong band around 492 cm^−1^ is assigned to the vibration of Ti-O bonds in TiO_6_ octahedral layers^15^. The weak absorption of HTO at 927 cm^−1^ is attributable to the bending mode of hydroxyl groups^30^. In the case of the H_2_O_2_ treated HTO, two clearly peaks at 890 and 706 cm^−1^ are observed. The peak at 891 cm^−1^ is broad and asymmetric, suggesting that it consists of several overlapping bands. By Gaussian curve fitting, it can be fitted to two peaks at 890 and 927 cm^−1^ which are assigned to the O-O stretching vibration and the bending mode of hydroxyls groups, respectively^30^,^31^,^32^. The apparent peak at 706 cm^−1^ can be ascribed to the vibration of the Ti-O-O bond^32^. Although the very weak peak at 706 cm^−1^ is detected in spectrums of HTO, we do not consider the presence of Ti-O-O bonds because of the inexistence of O^1−^ the in HTO crystal. Therefore, combining with XPS results, we can confirm the existence of the Ti-O-O coordination bonds in the H_2_O_2_ treated HTO crystal.

**Figure 4:**
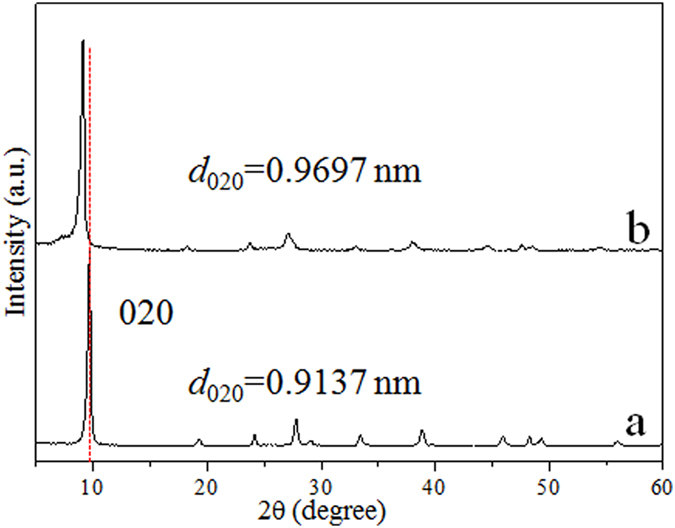
XRD patterns of the HTO crystals (**a**) before and (**b**) after treatments H_2_O_2_ solution, respectively.

HTO crystal is an open structure compound which consisted of TiO_6_ octahedrons layers and interlaminar hydrated H^+^ ions. The (020) crystal plane of the HTO crystal corresponds to TiO_6_ octahedron layers of HTO, its spacing is 0.9137 nm (Fig. 4a). After the HTO crystal is treated by H_2_O_2_, it still remains the fine crystallinity, whereas its 020-plane spacing increases to 0.9697 nm (Fig. 4b). This indicates that the larger radius H_2_O_2_ molecules than H_2_O enter into the TiO_6_ octahedron interlayers of HTO. Combining with XPS and FTIR results, it is considered that a mass of Ti-O-O coordination bonds are formed in the TiO_6_ octahedron interlayers (inside HTO crystal), not only on its surface. Above results indicate that the peroxide layered titanium oxide HTO crystal is successfully prepared through a simple treatment of HTO in H_2_O_2_ the solution.

**Figure 5:**
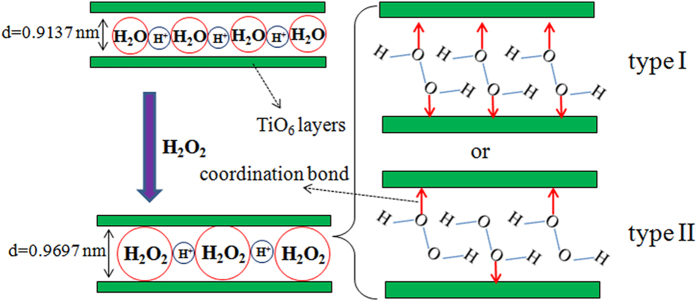
Formation mechanism of the peroxide layered titanium oxide HTO crystal from the layered titanium oxide HTO crystal.

On the basis of the above results, we propose a formation mechanism of the peroxide layered titanium oxide HTO crystal from the layered titanium oxide HTO crystal, as shown in Fig. 5. The mechanism of formation of the peroxide HTO crystal consists of the displacement reaction and *in situ* coordination reaction. When the HTO crystal comes in contact with the H_2_O_2_ molecules, the H_2_O_2_ molecules easily intercalate with TiO_6_ interlayer via H_2_O_2_/H_2_O exchange, resulting in the increase of TiO_6_ layers spacing (Fig. 4). At the same time, due to the strong Ti(IV) coordination ability of H_2_O_2_, the H_2_O_2_ molecules straightaway *in situ* coordinate with Ti within TiO_6_ octahedron in the interlayers (Fig. 3). Although we cannot accurately decide which coordination type (type I, type II in Fig. 5 or others) in the interlayers, it is affirmed that the peroxide layered titanium oxide HTO crystal with containing Ti-O-O coordination bonds in TiO_6_ interlayers is formed. The mechanism described above suggests that the titanium oxide crystal with open structure can be used as precursor for the preparation of the peroxide titanate.

**Figure 6:**
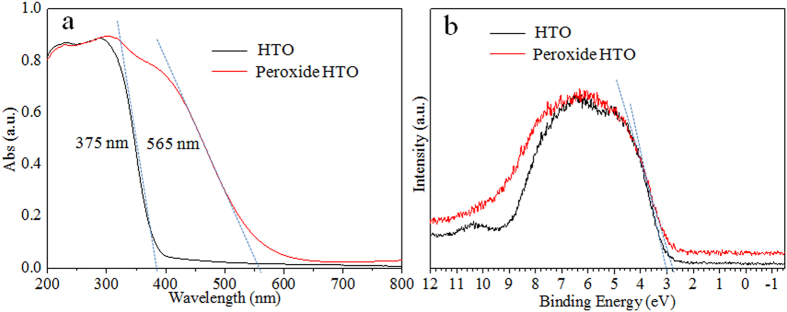
UV–vis diffuse reflectance spectra (**a**) and Valence band XPS spectra (**b**) of the HTO crystal and the peroxide HTO crystal.

Figure 6a shows UV-vis diffuse reflectance spectra of the HTO crystals before and after treatments with H_2_O_2_, respectively. In the case of HTO crystals, the absorption edge appears near 375 nm corresponding to the band gap of 3.3 eV. It is interesting that the peroxide HTO sample presents a red-shift absorption edge of 565 nm corresponding to the band gap of 2.2 eV, which is consistent with its yellow color appeared (Fig. 1b). This indicates that the presence of Ti-O-O bond contributes to the band gap narrowing of the layered HTO crystal.

**Figure 7:**
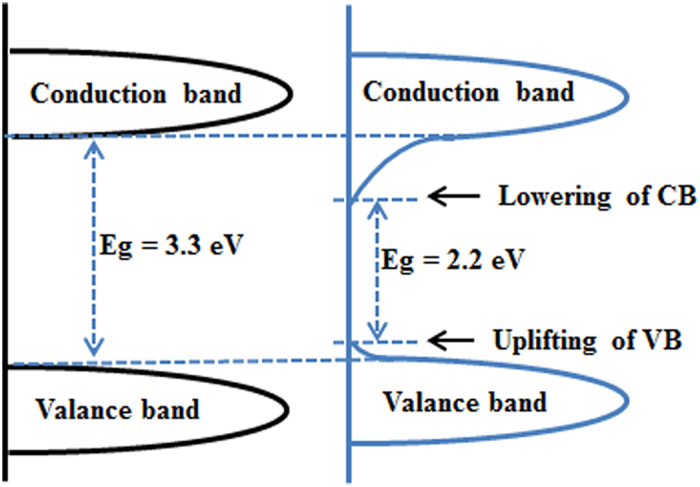
Schematic illustration of the density of states of the HTO crystal and the peroxide HTO crystal.

The band gap reduction could occur by the formation of mid gap band states either above the valence band (VB) or below the conduction band (CB) overlapping with the respective band. To understand the effect of Ti-O-O coordination bond on the HTO band gap narrowing, the VB XPS patterns of both the pure HTO and the peroxide HTO were recorded in Fig. 6b. The VB maximum of HTO is observed at 3.0 eV. For the peroxide HTO, the VB maximum, which is noted at 2.8 eV, would not have as substantial a change. Combining with the band gap of the pure HTO (3.05 eV) and the peroxide HTO (1.93 eV) from optical measurements, the CB minimum of the pure HTO and the peroxide HTO would occur at about −0.05 and 0.87 eV, respectively. It is suggested that the Ti-O-O coordination bond give rise to lower the CB minimum for HTO. A schematic illustration of the density of states the pure HTO and the peroxide HTO is shown in Fig. 7.

Since the yellow showed of peroxide HTO crystal is caused by the presence of the abundant Ti-O-O coordination bonds in the interlayers, we investigate the stability of peroxide sample to temperature, light and acid/base via the color change and structure of sample. The peroxide HTO displays the yellow color in the water with the pH value of 1~10, but turns white when the pH values are above 10. This indicates that the Ti-O-O coordination bonds is unstable and easily decompose under the alkaline condition. According to the XRD patterns of samples obtained after the heat-treatment of the peroxide HTO at different temperature (Supplementary Fig. S3), it is found that after the heat-treatment at 80 °C for 5 h, the 020-plane spacing of the sample is still 0.9697 nm and its color remains yellow. When at 100 °C for 5 h, the 020-plane spacing of sample decreases to 0.9459 nm and its color changes into the slight yellow, suggesting the disintegration of parts of Ti-O-O coordination bonds within the interlayers. This result is consistent with the TG measurements (Supplementary Fig. S4). Up to 200 °C, the color of sample becomes white and the layered structure is destroyed. It is implied that the peroxide sample has a fine stability below 80 °C. In addition, it is found that the peroxide sample irradiated by visible light still presents the yellow color, but the color of sample irradiated by UV light turns into white and both the crystallinity and 020-plane spacing decrease (Supplementary Fig. S5). It is indicated that the Ti-O-O coordination bonds can be disintegrated under the UV light irradiating. In fact, when our as-obtained peroxide layered titanate acid is reserved for half a year at condition of room temperature and natural light, it still has no change. Above these results conclude that the Ti-O-O coordination bonds within the peroxide HTO crystal are comparatively steady.

**Figure 8:**
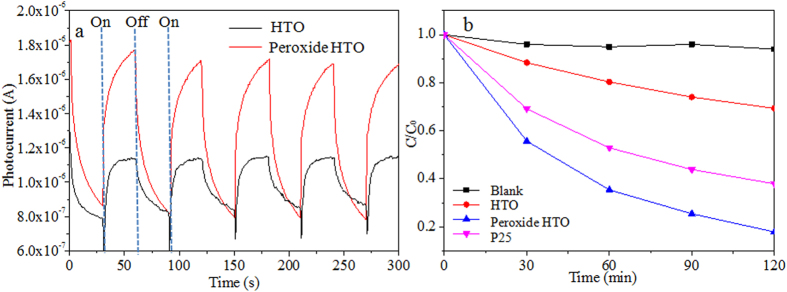
Photocurrent versus time measurements (**a**) and Visible light photocatalytic degradation (**b**) of the HTO crystal and the peroxide HTO crystal.

To investigate the photo responses of the HTO crystals before and after treatments with H_2_O_2_, the photocurrent transient response measurement was carried out under illumination with several cycles of 30 s interval light on or off in Na_2_SO_4_ aqueous solutions. As shown in Fig. 8a, a clear comparison of the I-t curves for samples under visible light irradiation with the same wavelength range applied in the photocatalytic reactions. Conspicuously, the photocurrent value of the peroxide HTO sample rapidly climbs up to a high current level when the light turns on and it presents a sharp decrease with the irradiation turning off. While it also returns to a constant value when the light turns on again. Similar phenomenon occurs upon HTO during the on-off irradiation process. However, it is noticeable that the photocurrent density of the peroxide HTO electrode (ca. 1.8 μAcm^−2^) is about two times higher than that of pristine HTO (ca. 1.1 μAcm^−2^). Thus, such results can further validate that the introduction of Ti-O-O coordination bonds in HTO crystal can promote the separation efficiency of the photo induced electron-hole pairs and a relatively lower recombination rate under visible-light^33^.

During the photodegradation of RhB over the peroxide HTO crystal under visible light illumination, it is seen clearly that the absorbance of RhB at the maximum absorption wavelength (551 nm) is gradually decreased with the prolongation of the irradiation time (Supplementary Fig. S6), which is because the chromophoric structure of the dye is destroyed. Figure 8b shows the photocatalytic performance (C/C_0_) versus visible light irradiation time of samples for the degradation of RhB. The HTO sample displays the degradation efficiency of about 15% after irradiation for 90 min. Nevertheless, it is surprising that the peroxide HTO crystal takes about 90 min to reach the degradation efficiency of about 85%. And the peroxide HTO crystal still keeps about 85% of the original efficiency after the cyclic degradation of five times, which forecloses the possibility of -O-O- oxidizing the RhB. For comparison, the commercial TiO_2_ (P25) presents degradation efficiency of about 50% for RhB after irradiation for 90 min.

In addition, the visible light photocatalytic activity of oxygen evolution for the peroxide HTO crystal is evaluated under visible light irradiation (>400 nm). Figure S7 shows the O_2_ evolution curves of the peroxide HTO crystal and the HTO crystal. It can be observed clearly that the peroxide HTO crystal has the visible light photocatalytic activity for water splitting into oxygen (2 μmol·g^−1^), but the HTO crystal has not. This is because that the bandgap of the HTO crystal too wide (3.05 eV), the photoproduction electronic and hole cannot be generated under visible light irradiating, resulting in that the water cannot be oxidized. In a word, the higher photocatalytic performance of the peroxide HTO crystal under visible light condition can be attributed to the narrowed band gap generated from the lower CB maximum (Fig. 7) and the higher separation efficiency of the photoinduced electron-hole pairs (Fig. 8a). Therefore, it is indicated that the existence of the Ti-O-O coordination bonds can markedly enhance the visible light photocatalytic performance of the HTO crystal.

## Conclusions

The peroxide HTO crystal can be prepared by the simple treatment for the layered HTO crystal in the H_2_O_2_ solution. A mass of Ti-O-O coordination bonds are formed in the TiO_6_ octahedron interlayers except on its surface, which results in the decrease of the band gap from 3.05 eV (HTO) to (1.93 eV the peroxide HTO). Combining with VB maximum of HTO (3.0 eV) and the peroxide HTO (2.80 eV) from VB XPS measurements, the CB minimum of HTO and the peroxide HTO occur at about −0.05 and 0.87 eV, respectively. Visible light induced photocatalytic degradation of RhB and splitting water into oxygen results further confirms the improved photocatalytic performance of the peroxide HTO crystal. This study suggests that the Ti-O-O coordination bonds can effectively enhance the visible light-induced photoactivity for the layered titanium oxide.

## Methods

The starting material of the layered titanium oxide H_1.07_Ti_1.73_O_4_ nH_2_O (HTO) with platelike particle morphology was prepared using the method described in the literature^34^. Peroxide HTO were synthesis by mixing 0.5 g HTO and 50 ml H_2_O_2_ (30% in water) under magnetic stirring for 5 min. Then the sample was washed with distilled water, and dried at room temperature to obtain the peroxide HTO.

The samples were characterized using a powder X-ray diffractometer (XRD, Rigaku D/max-2200PC) with Cu Kα (λ = 0.15418 nm) radiation, field emission scanning electron microscopy (FE-SEM, Hitachi S-4800). X-ray photoelectron spectroscopy (XPS) measurements were done on an Axis Ultra XPS instrument with an Al Kα source. The Fourier transform infrared (FTIR) spectra were measured in Bruker infrared spectrometer (VERTE70) with the KBr disk technique. The UV-vis absorption spectra were recorded on a UV/vis/NIR Spectrophotometer (LAMBDA950, PerkinElmer). The transient photocurrent responses were performed using CHI660E electrochemical station (Shanghai Chenhua, China).

The photocatalytic performances of samples were evaluated by degradation of rhodamine B (RhB), using 1000 W xenon lamp irradiation as light source. In each experiment, 50 mg of samples were added into the RhB solution (50 mL, 10 mg·L-1). The suspensions were magnetically stirred in dark for 60 min to ensure the establishment of an adsorption-desorption equilibrium. Then, the solution was exposed to the xenon irradiation under magnetic stirring. At different irradiation time intervals, 6 mL of the solution was collected with centrifugation. The concentration of the remnant dye in the collected solution was monitored by UV-vis spectroscopy (Unico UV-2600) each 30 min. The photocatalytic oxygen evolution experiments were performed in a 300 mL quartz reactor at 4 °C which was connected with a low-temperature thermostat bath. PLS-SXE 300UV Xe lamp with a UV-cutoff (≥400 nm) filter was used as the light source. In the oxygen evolution experiment, 50 mg of photocatalyst powder was suspended in a 100 mL of aqueous solution containing 0.05 M silver nitrate (AgNO3) solution as a sacrificial reagent. The amount of evolved Oxygen was determined by gas chromatog-raphy (Beifen 3420A, high purity Argon as a carrier gas, 99.999%) equipped with a thermal conductivity detector.

## Additional Information

**How to cite this article**: Kong, X. *et al*. Ti-O-O coordination bond caused visible light photocatalytic property of layered titanium oxide. *Sci. Rep.* **6**, 29049; doi: 10.1038/srep29049 (2016).

## Supplementary Material

Supplementary Information
