# Supplementary material for: Ti-O-O coordination bond caused visible light photocatalytic property of layered titanium oxide
Source: Sci Rep. 2016 Jun 28;6:29049. doi: 10.1038/srep29049 (PMC4924090; doi:10.1038/srep29049)
Supplement: Supplementary Information [file srep29049-s1.pdf]

Supplementary information

Ti-O-O coordination bond caused visible light photocatalytic property of layered titanium oxide

Xingang Kong[a*], Chaobin Zeng[a], Xing Wang[a], Jianfeng Huang[a*], Cuiyan Li[a], Jie Fei[a], Jiayin Li[a], Qi Feng[b]

[a] School of Materials Science and Engineering, Shaanxi University of Science and Technology, Weiyang, Xi'an, Shaanxi 710021, PR China

[b] Department of Advanced Materials Science, Faculty of Engineering, Kagawa University, 2217-20 Hayashi-cho, Takamatsu-shi, 761-0396 Japan

*Author to whom correspondence should be addressed. E-mail: yezhu_1983@163.com, huangjfsust@126.com

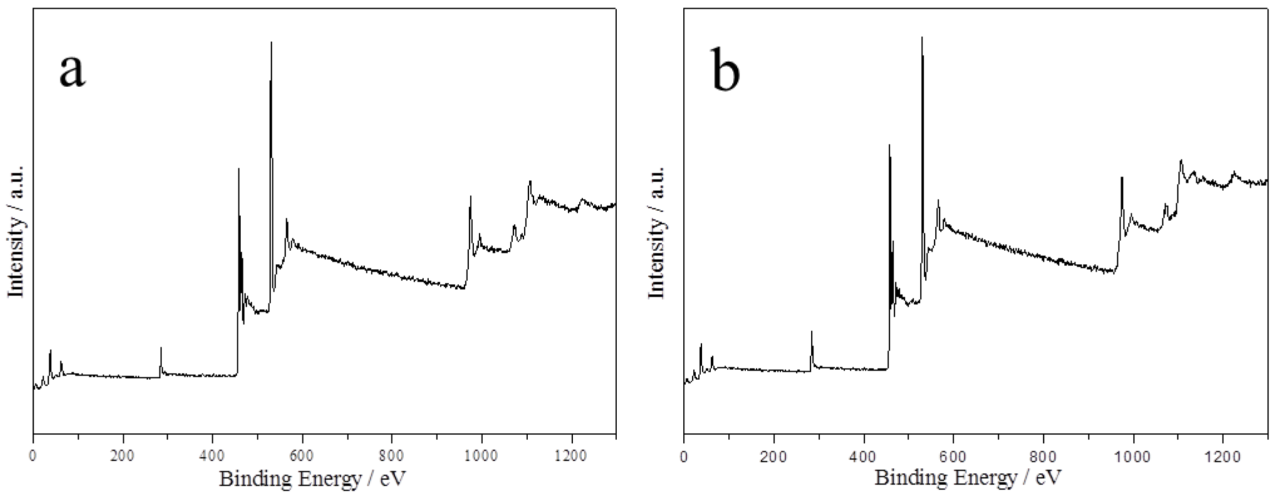

Fig. S1 XPS survey spectrum of (a) the HTO crystal and (b) the H_2O_2 treated HTO crystal.

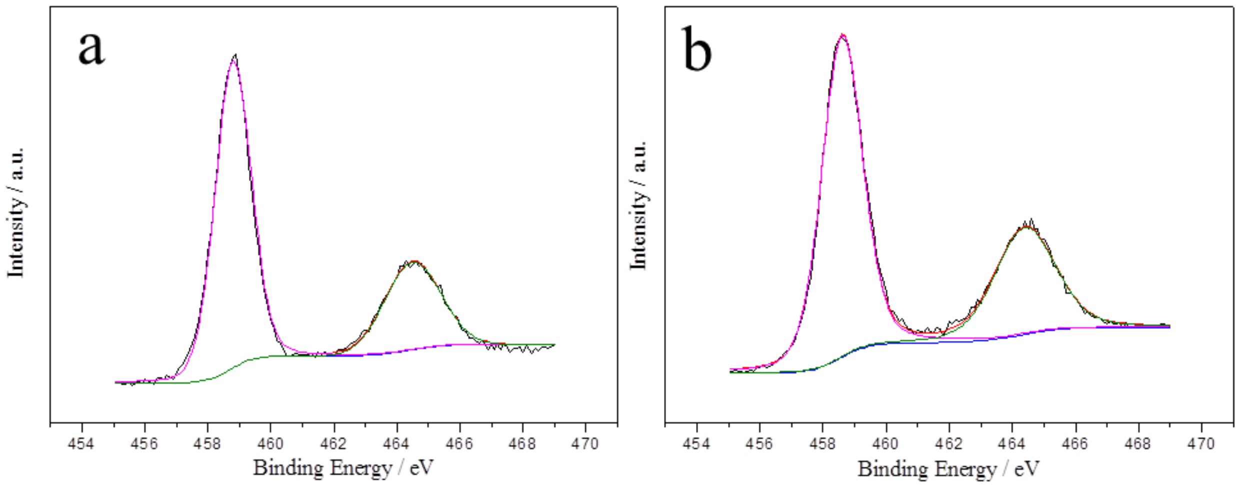

Fig. S2 XPS spectra of Ti 2p for (a)the HTO crystal and (b)the H_2O_2 treated HTO crystal.

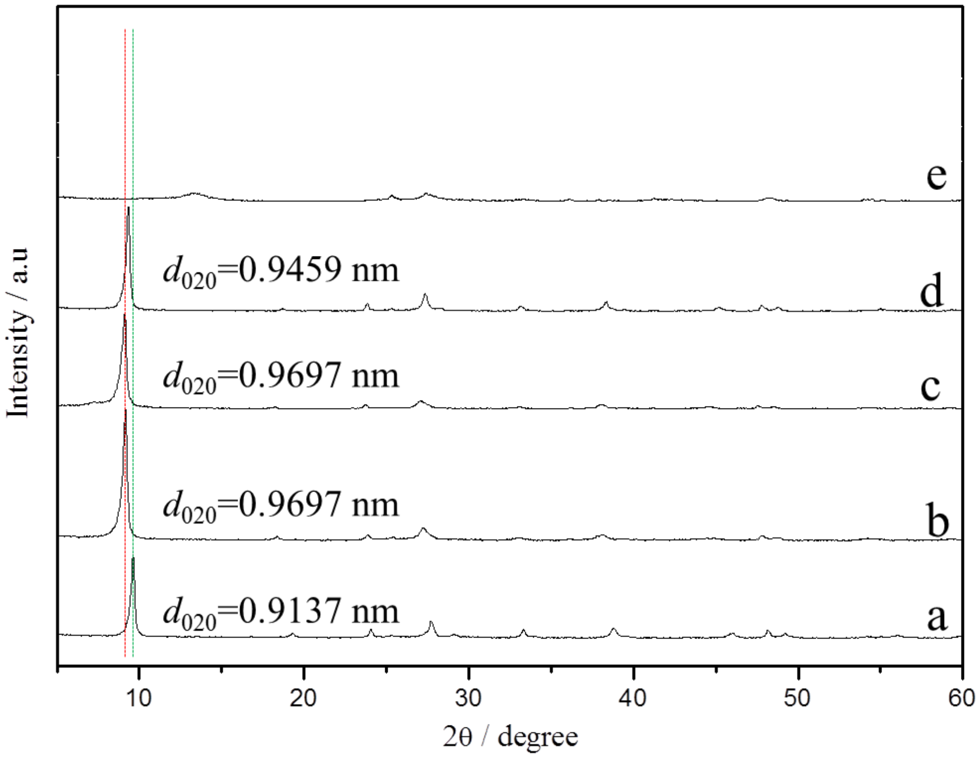

Fig S3 XRD patterns of sample obtained after the heat-treatment of the peroxide HTO crysyal at different temperature. (a) HTO, (b) 60 ^{o}C, (c) 80 ^{o}C, (d) 100 ^{o}C, (e) 200 ^{o}C.

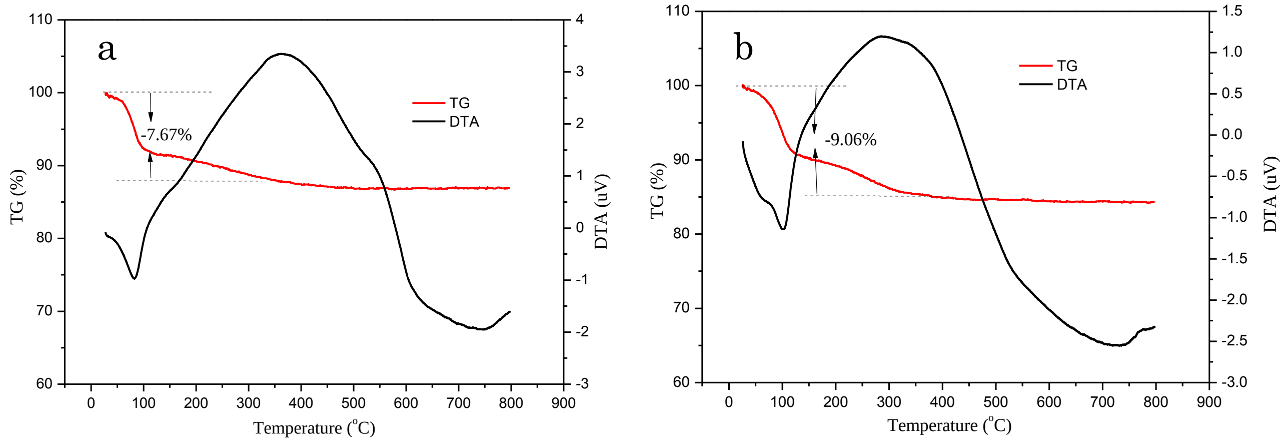

Fig S4 TG measurements of (a) HTO and (b) the peroxide HTO.

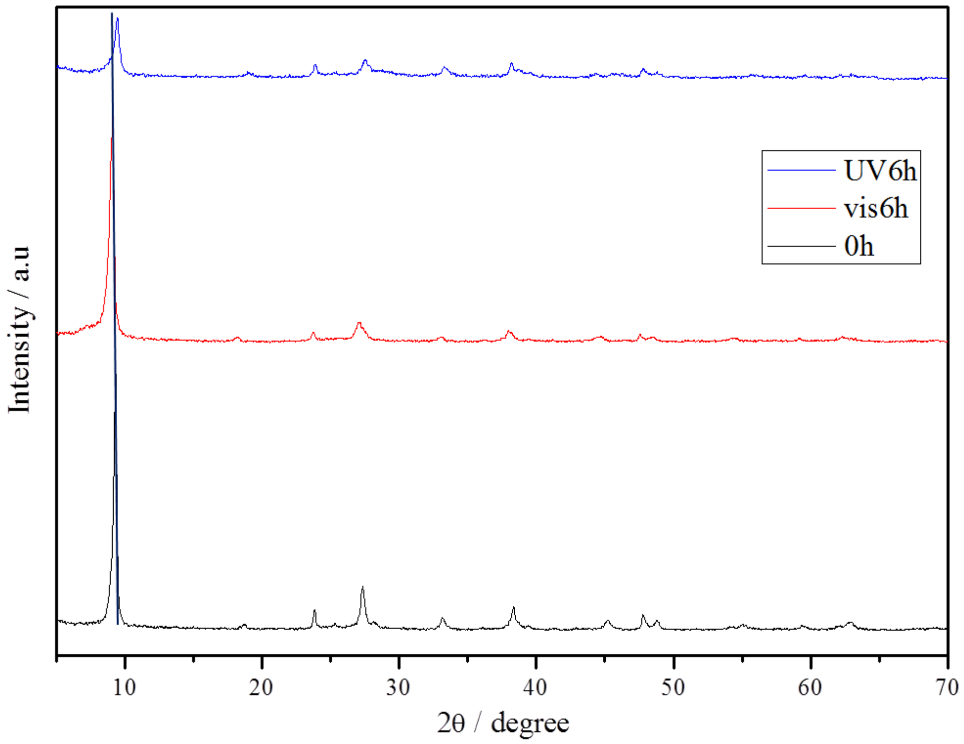

Fig. S5 XRD patterns of sample obtained after irradiating with UV and visible light for 6h, respectively.

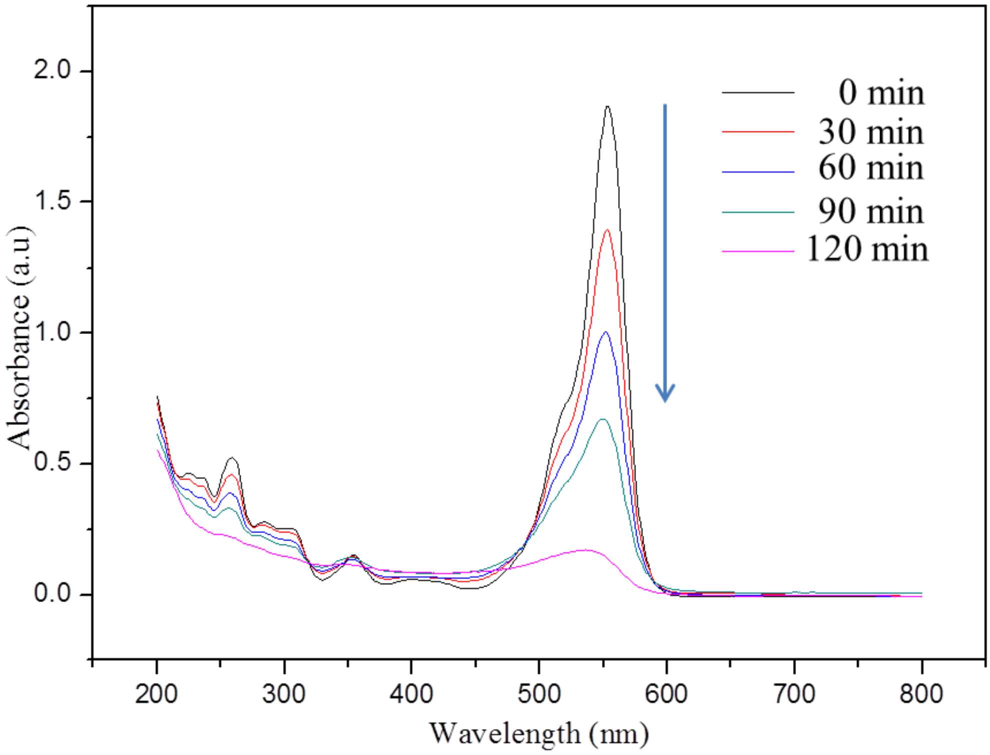

Fig. S6 Time-dependent UV-vis absorption spectra of the RhB solution in the presence of the peroxide HTO crystal.

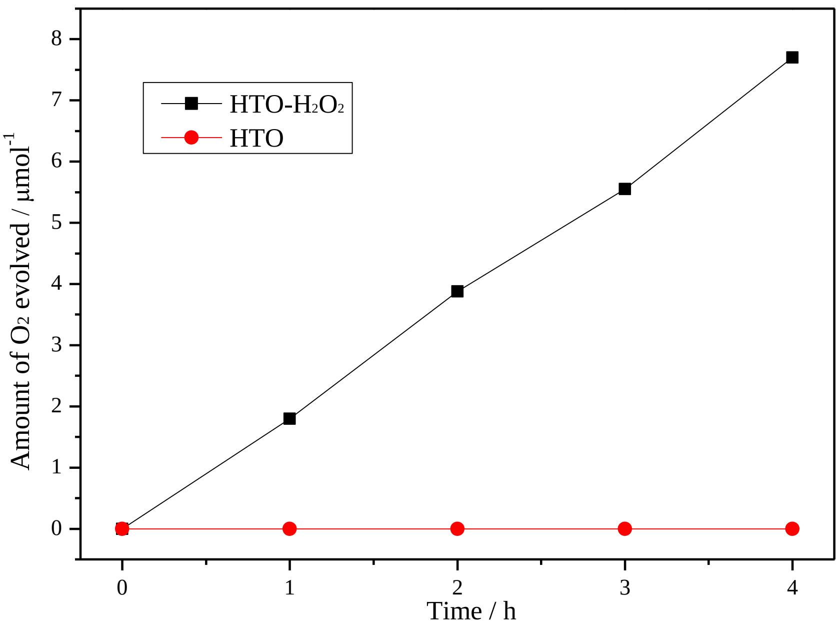

Fig. S7 Photocatalysts for water splitting into oxygen under visible light irradiation.
